# North American wintering mallards infected with highly pathogenic avian influenza show few signs of altered local or migratory movements

**DOI:** 10.1038/s41598-023-40921-z

**Published:** 2023-09-02

**Authors:** Claire S. Teitelbaum, Nicholas M. Masto, Jeffery D. Sullivan, Allison C. Keever, Rebecca L. Poulson, Deborah L. Carter, Abigail G. Blake-Bradshaw, Cory J. Highway, Jamie C. Feddersen, Heath M. Hagy, Richard W. Gerhold, Bradley S. Cohen, Diann J. Prosser

**Affiliations:** 1 Akima Systems Engineering, Herndon, VA USA; 2 Contractor to U.S. Geological Survey, Eastern Ecological Science Center, Laurel, MD USA; 3 College of Arts and Sciences, Tennessee Technological University, Cookeville, TN USA; 4 U.S. Geological Survey, Eastern Ecological Science Center, Laurel, MD USA; 5 Southeastern Cooperative Wildlife Disease Study, College of Veterinary Medicine, University of Georgia, Athens, GA USA; 6 Tennessee Wildlife Resources Agency, Nashville, TN USA; 7 U.S. Fish and Wildlife Service, National Wildlife Refuge System, Stanton, TN USA; 8 University of Tennessee College of Veterinary Medicine, Knoxville, TN USA; 9 Present Address: Bay Area Environmental Research Institute and NASA Ames Research Center, Moffett Field, CA USA

**Keywords:** Ecology, Animal migration, Ecological epidemiology, Wetlands ecology, Ecology, Animal migration, Ecological epidemiology, Wetlands ecology, Animal behaviour

## Abstract

Avian influenza viruses pose a threat to wildlife and livestock health. The emergence of highly pathogenic avian influenza (HPAI) in wild birds and poultry in North America in late 2021 was the first such outbreak since 2015 and the largest outbreak in North America to date. Despite its prominence and economic impacts, we know relatively little about how HPAI spreads in wild bird populations. In January 2022, we captured 43 mallards (*Anas platyrhynchos*) in Tennessee, USA, 11 of which were actively infected with HPAI. These were the first confirmed detections of HPAI H5N1 clade 2.3.4.4b in the Mississippi Flyway. We compared movement patterns of infected and uninfected birds and found no clear differences; infected birds moved just as much during winter, migrated slightly earlier, and migrated similar distances as uninfected birds. Infected mallards also contacted and shared space with uninfected birds while on their wintering grounds, suggesting ongoing transmission of the virus. We found no differences in body condition or survival rates between infected and uninfected birds. Together, these results show that HPAI H5N1 clade 2.3.4.4b infection was unrelated to body condition or movement behavior in mallards infected at this location during winter; if these results are confirmed in other seasons and as HPAI H5N1 continues to evolve, they suggest that these birds could contribute to the maintenance and dispersal of HPAI in North America. Further research on more species across larger geographic areas and multiple seasons would help clarify potential impacts of HPAI on waterfowl and how this emerging disease spreads at continental scales, across species, and potentially between wildlife and domestic animals.

## Introduction

Infectious diseases associated with wildlife have emerged at increasing rates in the last 50 years, a trend that is linked to declines in biodiversity and changes in climate and land use^1–4^. Avian influenza viruses (AIVs) are one such emerging threat to wildlife, domestic animals, and potentially human health. Low pathogenic avian influenza viruses (LPAI) circulate endemically in wild waterfowl populations (ducks, geese, and swans; order Anseriformes) and generally cause little or no clinical disease^5^. However, since the 2.3.4.4 clade of the A/goose/Guangdong/1/1996 H5N1 lineage of highly pathogenic influenza (HPAI) emerged in 2010, it has caused substantial mortality in many sensitive wild bird populations and significant economic impacts to commercial poultry operations^6,7^. Outbreaks of HPAI have been concentrated in Eurasia, where these viruses are beginning to be independently maintained in wild birds and cause detrimental effects to many species^8,9^. In November 2021, the 2.3.4.4 clade was detected in North America for the first time since 2015^10^. It has since spread across the contiguous U.S. and Alaska, across 12 Canadian provinces and territories, and into Central and South America^11^. Given its pandemic potential in wild birds and poultry^9,12^, it is crucial to further understand how HPAI impacts wild bird health and how it spreads within and among wild bird populations.

Movement behavior of infected hosts drives the spread of infectious diseases and serves as an important indicator of an infection’s pathogenicity. For example, a pathogen that imposes an energetic cost can reduce infected hosts’ movement ability, thus reducing contact rates and limiting transmission. Infection with LPAI is sometimes associated with reduced movement in wild waterfowl at both local and migratory scales^13^, but just as often LPAI infection has no effect on waterfowl behavior^14^. However, HPAI viruses likely have stronger negative effects than LPAI viruses on waterfowl movement behavior. For example, a lesser scaup (*Aythya affinis*) infected with HPAI H5N1 in Maryland, USA in January 2022 exhibited reduced local movements and subsequent mortality (cause unknown); despite these reduced movements, this individual still could have contacted multiple uninfected birds while infected with HPAI H5N1^15^. Conversely, a white-faced whistling duck (*Dendrocygna viduata*) infected with a highly pathogenic strain of avian influenza (HPAI H5N2) in West Africa displayed similar movement patterns as uninfected conspecifics^16^. Laboratory studies also show wide variation in responses to HPAI infection across waterfowl species and individuals, including in viral pathogenicity and shedding rates^17–20^, which can be modulated by individuals’ previous exposure to HPAI and/or LPAI^21,22^. Each species’ unique relationship between HPAI infection and movement behavior likely influences its role in the dispersal of HPAI at local, continental, and global scales.

Among waterfowl, mallards (*Anas platyrhynchos*) and other dabbling ducks are the best-known reservoir species for AIVs^23^. Although mortalities have been reported in wild mallards infected with HPAI, including in the 2021–2022 North American outbreak^24^, most mallards experimentally infected with HPAI H5N1 in laboratory settings show few or no clinical signs despite shedding large quantities of virus^25–27^. Mallards are also the most abundant waterfowl species globally, are distributed across the Northern Hemisphere, and exhibit complex migratory patterns including within-population variation in migration propensity and distance, making them an important species for both dispersal and local maintenance of AIVs^28,29^. Finally, mallards are relatively adaptable to human activities and often occupy urban and agricultural areas^30,31^. Their abundance in anthropogenic landscapes makes mallards a potential source of spillover or spillback of AIVs between wild and domestic birds. However, despite their potentially important role for HPAI infection dynamics, we know little about how HPAI infection affects mallard movement behavior, and until now, have had no data on North American mallards’ movement responses to newly emerged HPAI H5N1.

In January 2022, we detected HPAI H5N1 in 11 wild mallards in Tennessee, USA. These are the first known detections of HPAI in wild waterfowl in the Mississippi Flyway during the 2021–2022 North American outbreak^24^. These mallards, which showed no signs of disease at capture, and 32 uninfected conspecifics were fitted with GPS transmitters that provided hourly locations. We used these data to compare local movement behavior and migration patterns between infected and uninfected individuals, and to identify spatio-temporal interactions between marked birds that could have resulted in HPAI transmission. We expected that HPAI H5N1 infection would have pathogenic effects on mallards, which would be reflected in reduced movement by infected mallards shortly after detection of the virus. We expected that this reduced movement would decrease contact rates and shared space use between infected and uninfected birds. We also hypothesized that energetic costs of infection could carry over to spring migration, which would be reflected in later, slower, and/or shorter-distance migration in mallards infected during winter, compared to those with no known history of HPAI infection. Finally, we compared mortality rates and body condition between infected and uninfected birds to understand whether infection with HPAI H5N1 had apparent energetic or fitness costs.

## Results

We captured 11 mallards infected with HPAI H5N1 and 32 that were not shedding any AIV in Tennessee, USA in January 2022. HPAI infection prevalence was 0.39 in females (*n* = 7/18), 0.16 in males (*n* = 4/25), 0.32 in juveniles (*n* = 7/22), and 0.19 in adults (*n* = 4/21). Prevalence of antibodies to the nucleoprotein of AIV was 0.57 overall (*n* = 23/40; antibody data were unavailable for three individuals) and 0.54 in HPAI-infected birds (*n* = 6/11); detection of antibodies could indicate either prior exposure to influenza (HPAI or LPAI) or seroconversion from a recent infection. No clinical signs of illness were observed at the time of capture.

### Local movements

**Figure 1:**
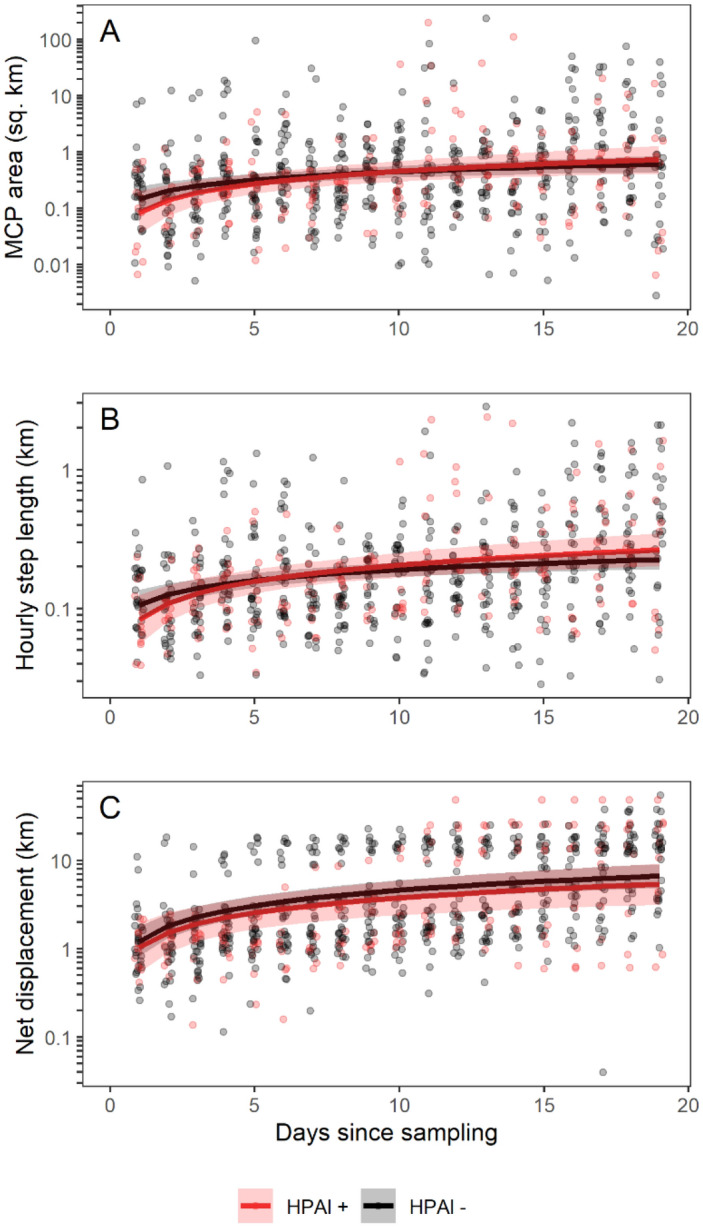
Local movement patterns are unrelated to infection with HPAI H5N1 clade 2.3.4.4b in 43 mallards (*Anas platyrhynchos*) sampled in Tennessee, USA during winter 2022. In each plot, points show raw data, lines show estimated means from a linear mixed-effects model, and shaded areas show 95% confidence intervals of the mean. Models also included terms for age, sex, and a temporal autoregressive term for each individual; plots show marginal values averaged across age and sex. For plots that show predictions conditional on random effects, see Fig. S1. (**A**) Area of a 100% minimum convex polygon (MCP), a measurement of space use. (**B**) Mean hourly step lengths, a measurement of overall movement. (**C**) Net displacement, i.e., mean daily distance from the first GPS fix, a measurement of dispersal from the capture site.

Local movement behaviors in the first 19 days following sampling were unrelated to HPAI infection status (Fig. 1, Tables S1–S3); the 19-day period of study was designed to include both active infection and recovery for HPAI-infected birds and ended before any tracked mallards initiated migration. On the first day following sampling, when differences between groups would be expected to be largest, the average area of a HPAI-infected mallard’s daily 100% minimum convex polygon (MCP) was 0.085 km^2^ (95% CI: 0.036–0.203), which was indistinguishable from that of the average uninfected mallard (mean: 0.148 km^2^, 95% CI: 0.087–0.250). Regardless of infection status, mallard space use increased following sampling and release, probably indicating temporary effects of capture or transmitter attachment and not infection on movement. We also found no difference in movement behavior by infection status for hourly movement distances or mean net displacement (Fig. 1B, C; Tables S2, S3). In a second set of models, we found no evidence that AIV antibody status (which could indicate either seroconversion from the current infection or from a prior infection) moderated the relationship between HPAI active infection and movement behavior (Table S4).

### Contact rates

We observed 375 interactions between pairs of mallards (i.e., a mallard was detected within 25 m of a known location of another bird within 65 min; Fig. S2), of which 80 (23%) were potential close or indirect HPAI contacts, i.e., an infected bird followed by an uninfected bird. When we compared this proportion to the expected frequency of contacts in the population, the observed proportion was in the 75th percentile of the randomized data, indicating no significant difference between the observed frequency of contacts and the expected frequency assuming birds were interacting independently of infection status.

**Figure 2:**
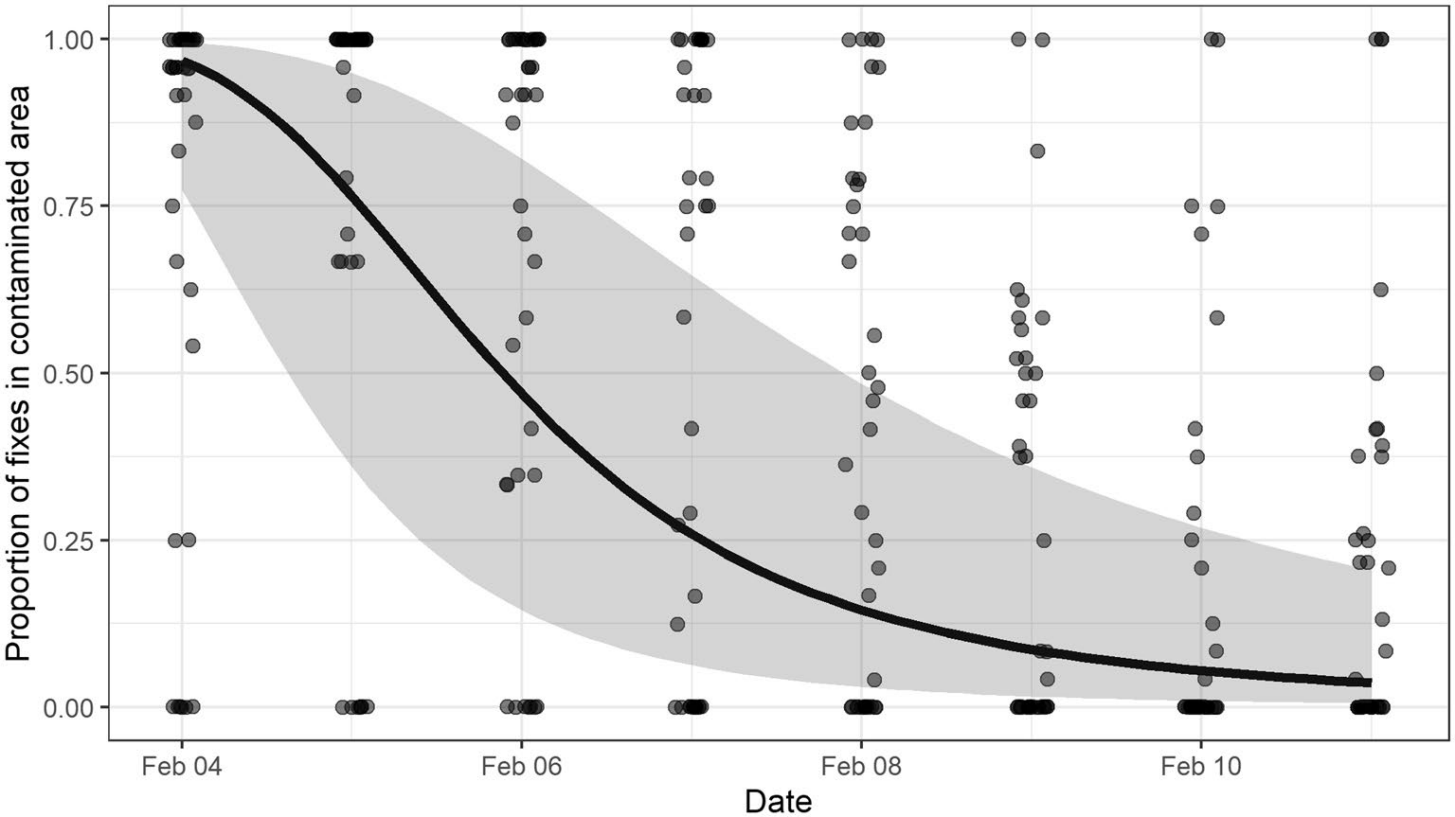
Time spent in the potentially HPAI-contaminated area declines prior to initiation of migration. The contaminated area was defined as the total area of all 95% utilization distributions of HPAI-infected mallards (*Anas platyrhynchos*) in the first four days following sampling. The proportion of time was calculated as the proportion of daily fixes for each mallard within the contaminated area. Points show raw data and are jittered to increase visibility. The line and shaded area show marginal means from a generalized linear mixed-effects model. The model also included terms for HPAI infection status, age, and sex; only sex was related to time spent in the contaminated area (Table S5, Fig. S3).

Infected birds used a cumulative total area of 6.9 km^2^ during the first four days following sampling; birds were likely to be shedding HPAI for at least four days after sampling, so we considered this area potentially HPAI-contaminated (hereafter “contaminated area”). All birds initially spent most of their time in the contaminated area, but use of this area declined as the winter progressed, at similar rates for infected and uninfected birds (Fig. 2, Table S5). Our model estimated that on the first day of measurement (February 4), tracked mallards spent > 90% of their time in the contaminated area, but this time decreased to < 5% by February 9. There was substantial variation among individuals; two individuals (6%) were never detected in the contaminated area while two others spent all their time in the contaminated area through the end of the study period (individual ID standard deviation = 5.085; AR1 correlation = 0.862). Males spent more time in the contaminated area than females (Table S5).

### Migration patterns

**Figure 3:**
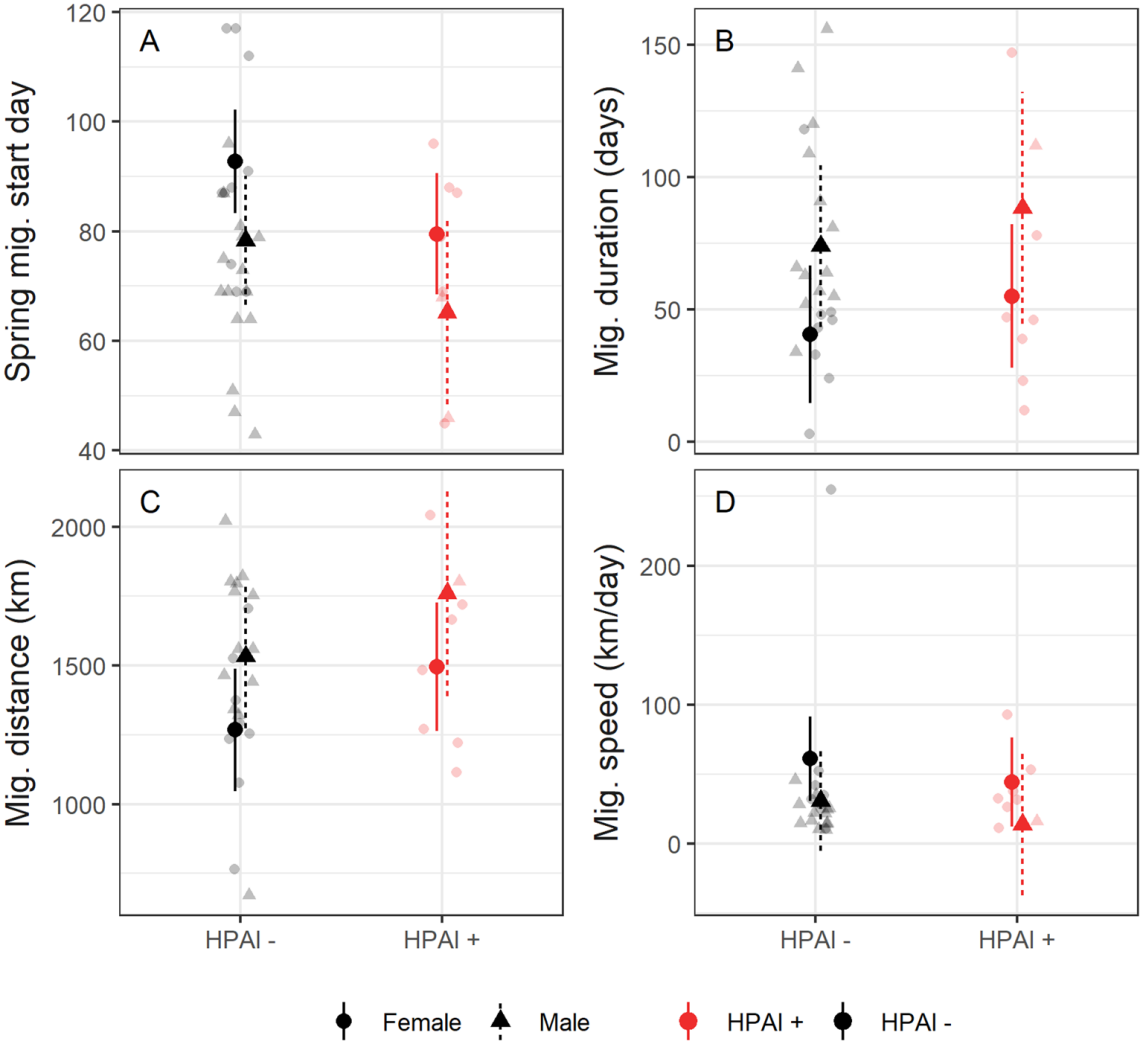
Relationships between HPAI infection status, sex, and migration patterns in mallards (*Anas platyrhynchos*). Each panel shows the estimated mean and 95% confidence interval of the mean from a linear model. Partially transparent points show raw data. Models also included a term for age; plots show values for juveniles. (**A**) HPAI-infected birds departed on spring migration slightly earlier than uninfected birds and males migrated earlier than females. The y-axis shows the day of year of spring migration initiation (day 80 = March 21). (**B**) The duration of migration was unrelated to infection status. (**C**) Migration distance was unrelated to infection status, but males migrated farther than females. (**D**) Migration speed was unrelated to infection status or sex.

We quantified migration patterns for birds with sufficient telemetry data to measure the beginning of spring migration (*n* = 35) and arrival at summer sites (*n* = 29); some birds lacked sufficient data due to mortality, lack of transmitter signal, or transmitter failure. The mean spring migration initiation date was March 15 for infected birds (*n* = 9) and March 20 for uninfected birds (*n* = 26). Infected birds departed slightly earlier than uninfected birds (13 days, 95% CI: 27 days earlier to 0.2 days later, R^2^ = 0.33; Fig. 3, Table S6) and males departed earlier than females (14 days, 95% CI: 1–28 days earlier).

The time between winter site departure and summer site arrival (i.e., migration duration) averaged 63 days for infected birds (*n* = 8) and 69 days for uninfected birds (*n* = 21). Our model indicated no difference in migration duration by infection status or age (Fig. 3, Table S6; R^2^ = 0.24). There was weak evidence that males migrated for longer than females (estimate: 33 days longer, 95% CI: 3 days shorter to 70 days longer).

The average migration distance was 1540 km for infected birds (*n* = 8) and 1445 km for uninfected birds (*n* = 21). Our model showed no evidence for a difference in migration distances in infected birds (difference: 228 km, 95% CI: 80 km shorter to 536 km farther, R^2^ = 0.17; Fig. 3, Table S6). We found no evidence for a difference in migration distance by age or sex.

The average migration speed for infected birds was 38 km/day and for uninfected birds was 36 km/day. We found no relationship between infection status and migration speed (estimate: 16 km/day, 95% CI: 24 km/day slower to 57 km/day faster, R^2^ = 0.14; Fig. 3, Table S6). We also found no evidence for differences in migration speed by age or sex.

We found no evidence that AIV antibody status was related to migration date, duration, distance, or speed (Fig. S4, Table S7).

### Body condition and mortality

We found no evidence for differences in body condition at capture between infected and uninfected birds (F_1,47_ = 0.073, p = 0.787) or for differences in survival by infection status, age, or sex. Model-estimated mortality was 0.38 for infected birds (*n* = 7; 95% CI: 0.01–0.77) and 0.33 for uninfected birds (*n* = 14; 95% CI: 0.05–0.62).

## Discussion

We detected infections with HPAI H5N1 clade 2.3.4.4b in 11 of 43 (26%) mallards sampled in Tennessee, USA, during January 2022. These detections represent some of the earliest detections in live birds during the ongoing HPAI outbreak in North America, which has severely impacted wild bird health (e.g., colonially nesting seabirds) and poultry production^11^. Collectively, our analyses show that HPAI infection in wild mallards during winter had no detectable effects on movement behavior at local (within 19 days) or migratory scales or on body condition or survival. Importantly, we observed shared space use between infected and uninfected birds on the wintering grounds as well as extensive movement of infected birds on the wintering grounds (up to 50 km from the capture site) and during migration. Together, these results suggest that tolerance of HPAI H5N1 infection could promote transmission within this wintering mallard population and beyond, including to other species and geographic areas.

Our finding that HPAI-infected and uninfected birds migrated similarly suggests that mallards had the potential to be effective dispersal agents for this emerging virus during its initial introduction to North America in winter 2021–2022. In general, host-parasite combinations where pathogenicity is low or tolerance is high should be associated most strongly with long-distance pathogen dispersal^32^, especially for migratory species^33^. Although the expected duration of infection with this clade of HPAI H5N1 can be up to 14 days (in experimentally-exposed immunologically naïve mallards^27^) and most migrations began more than 14 days after sampling, the shared space use that we observed suggests potential ongoing transmission during winter. Thus, we strongly suspect that many birds could be actively infected at the time of their migration. In addition, all birds either completed their migrations or made a stopover in less than 14 days (Fig. S5), thus providing a potential mechanism for long-distance spread of this pathogen^34^. However, infection statuses of all marked birds were unknown at the time of migration. It is possible that active and recent HPAI infection affects migration behavior, but that we could not detect these effects because recovery and transmission occurred between the time of sampling and initiation of migration. Nevertheless, our data and analyses show no relationship between infection and movement behavior in the week following sampling, or between infection and body condition, collectively suggesting that HPAI H5N1 infection had minimal negative effects on health or behavior in these wild North American mallards.

Laboratory studies show that HPAI infection often has minimal or no effects on duck health or behavior^27,35,36^, and that in experimental settings, mallards can shed high concentrations of HPAI H5N1 relative to other duck species^26^. Likewise, we found no differences in body condition or mortality between infected and uninfected birds in this wild population, even though natural settings exhibit higher variability in food availability^37,38^, body condition^39,40^, social interactions^41^, previous AIV exposure, and influenza viral loads in the environment^41^ than laboratory settings, all of which could influence the dynamics and pathogenicity of influenza infection. Still, infections with the same pathogen can differ in their pathogenicity across individuals and across time, depending on body condition, time since infection, behavior, or infection history^39,42–44^; if the most negatively affected birds are more likely to die or “hunker down,” they would not have been sampled, thus potentially biasing our sample towards individuals that are tolerant of HPAI infection. We also found no evidence that AIV antibodies mediated the effects of HPAI infection on movement behavior. Antibody prevalence is relatively high during winter^45^, which could have limited our ability to detect subtle changes in behavior of infected mallards; this protective benefit of prior exposure could differ at other times of year or in groups of immunologically naïve birds (e.g., juveniles), which could alter infection-movement relationships. A combination of experimental, observational, and theoretical studies across more species and seasons is necessary to fully understand how immunology and the environment interact to determine the impacts of influenza infection on wild bird behavior and health.

Mechanistic models are important tools for understanding the maintenance, dispersal, transmission, and reassortment of influenza viruses^46,47^, but often face uncertainties in parameter values (e.g., HPAI pathogenicity) or model structures (e.g., HPAI transmission routes). This study can inform several important parameters for these models. First, in North American wintering mallards with some prior AIV exposure, infection with HPAI H5N1 is unrelated to movement distances at local or migratory scales based on our fine-scale location data; therefore, modeling movement as homogeneous across infection statuses could be a reasonable assumption in mechanistic models. Second, mallards in our study contacted one another independent of infection status, but shared space use between birds declined over the course of the winter, probably coincident with increases in movement and changes in habitat selection and availability as the hunting season ended and preparation for migration began^48^. This pattern suggests that, while contact rates and contact with virions in the environment might be homogeneous within a population, they might vary within seasons. HPAI was also detected concurrently in heterospecific birds at the same refuge (R. Gerhold, unpubl. data), which could further contribute to environmental contamination. Modeling these spatio-temporal patterns in environmental transmission will require more complex functions than assuming that all birds are equally likely to encounter influenza virus in the environment. These results can inform more realistic models that more accurately predict the mechanisms of HPAI transmission and dispersal.

The current HPAI H5N1 outbreak in North America has affected over 47 million domestic poultry in the United States and threatens some wild bird species of conservation concern, including seabirds and raptors^11,24,49,50^. As this outbreak continues, wildlife managers and farmers must adapt their practices to prevent influenza infection in these sensitive species. Our results suggest that mallard populations—which are important culturally as a game species and for wildlife viewing^51^—might not be substantially impacted by the ongoing outbreak, at least for wintering mallards with prior exposure to AIV infected with the genotype of HPAI circulating in North America in January 2022. However, reduced wetland availability, as has been observed over the last century^52^, can promote disease transmission within wild waterfowl populations by increasing local densities, contact rates, and probabilities of environmental transmission^53^. Waterfowl densities at these and other state- and federally-owned waterfowl refuges can be high^54,55^, meaning that contacts and shared space observed in our study represent only a small fraction of potential direct and environmental transmission among the entire (mostly unmarked) population. As these mallards move locally and northwards on their spring migration, they travel through agricultural areas^56^ and share stopover sites with other waterfowl species^57^. We therefore expect that, because of their apparent tolerance to infection and gregarious behavior, wild mallards (and potentially other waterfowl) are important for the epidemiology of HPAI H5N1 in North America. However, because influenza viruses are constantly evolving and some strains exhibit higher pathogenicity than others^7,58,59^, it is critical to continue to monitor the effects of HPAI H5N1 across larger samples of multiple wildlife species, especially as the virus continues to reassort with North American-origin LPAI. More broadly, these results highlight that interspecific variation in behavior and responses to an emerging infectious disease can impact how these diseases spread, how long they persist, and their potential impacts on wildlife, domestic animal, and human health.

## Methods

### Study area, capture, and sampling

We captured male and female mallards using rocket nets at Lake Isom National Wildlife Refuge (NWR; 36.3049° N, − 89.4173° W) on 24, 25, 30, and 31 January 2022 (*n* = 20, 8, 5, and 10 individuals, respectively^60^. Lake Isom NWR was established in 1938 as Tennessee’s first NWR and maintains a diversity of managed wetlands including croplands, forested wetlands, and a large ~ 150 ha shallow-water lake that is seasonally dried, mechanically manipulated, and flooded during winter to provide moist-soil vegetation and seeds for wintering waterfowl. Lake Isom NWR hosts nearly 40,000 ducks on average in January (January 2022 aerial estimate = 36,834^55^).

We banded all captured mallards with U.S. Geological Survey aluminum tarsal bands and determined sex and age based on cloacal inversion, wing plumage, and bill color^61^. We aged ducks as juvenile (second year) or after adult (after second year). We measured weight (± 0.10 g) and wing cord length (± 1 mm) for all individuals. We collected oropharyngeal and cloacal swabs of all individuals and placed paired swabs into 2 mL viral transport medium (VTM)^62^. We also extracted ≤ 3 mL of blood from the brachial artery for each individual and separated the serum fraction. Swabs and sera were stored at − 80 °C until sent for virologic testing and additional analyses at the University of Georgia (Athens, GA, USA).

We attached 20-g solar rechargeable and remotely programmable Global Positioning System-Global System for Mobile (GPS-GSM) transmitters (OrniTrack; Ornitela, UAB Švitrigailos, Vilnius, Lithuania) to birds weighing ≥ 1 kg to ensure deployment package remained below recommended body weight limits (3–5%^63^). We attached transmitters using dorsally-mounted body harnesses made of automotive moisture-wicking elastic ribbon^64^. Completed harnesses had two body loops knotted and sealed with cyanoacrylic glue above the keel and across the abdomen^64^. Total package of GPS-GSM transmitter and harness weighed ~ 22 g. Transmitters were remotely programmed to record hourly locations and were not synchronized among individuals. Calibration data on this tag model indicates median location error of < 25 m. We used all available telemetry data from AIV-sampled birds from the first capture (24 January 2022) until we began analysis (27 October 2022)^65^.

All duck capture, handling, and sampling procedures were approved by and carried out in accordance with Tennessee Technological University’s Institutional Animal Care and Use Committee (protocol #19-20-0020) and authorized under Federal Banding Permit #05796. This study complies with the relevant portions of the ARRIVE guidelines for observational studies.

### Influenza lab methods

We attempted virus isolation on all swab samples by inoculating a total 1mL of VTM into the allantoic cavities of three 9–11 day-old embryonated chicken eggs^66^ and incubating at 37 °C for 120 h. Amnioallantoic fluid was collected and tested by hemagglutination assay^67^. RNA was extracted from amnioallantoic egg fluids for all putative virus isolation-positive samples using the QIAamp viral RNA mini kit (Qiagen Inc.; Germantown, MD, USA) following manufacturer recommendations, and screened for the matrix gene of influenza A virus in real-time reverse transcription polymerase chain reaction (rRT-PCR) as previously described^68^. Influenza A-positive samples were further screened for 2.3.4.4 HP H5 via rRT-PCR; suspect positives from this assay were sent to the United States Department of Agriculture National Veterinary Services Laboratory, Ames, Iowa for confirmation. A positive virus isolation result indicates active shedding of influenza at the time of capture.

Because no birds displayed visible indications of illness, laboratory testing was completed after capture and release, meaning that infection statuses were unknown at time of release.

Serum samples were tested for the presence of AIV antibodies by commercial blocking enzyme-linked immunosorbent assay (bELISA, IDEXX Laboratories, Westbrook, ME) as described by the manufacturer. An initial serum-to-negative control (S:N) absorbance ratio < 0.5 represents the cutoff threshold recommended by the manufacturer, so we considered samples with an S:N ratio > 0.5 to be positive. A positive bELISA result represents the presence of antibodies to AIV, which indicates prior infection with any AIV (HPAI or LPAI). Influenza antibodies are estimated to be detectable for 6 months–1.5 years^69–71^ but usually peak within 3 weeks of infection^69,70^.

### Data analysis

#### Local movements

We analyzed daily movement patterns within 19 days of capture to determine whether movement behavior differed between HPAI-infected and uninfected birds, beginning at the time of capture and ending after presumed recovery from infection (≤ 14 days;^27^). We expected that, if HPAI infection affected local movement behavior, infected and uninfected birds would move differently in the first few days following sampling, but any differences in movement would no longer be observed by the end of the 19-day window. One mallard started migrating 20 days after capture, so we used a 19-day window to include non-migratory movements only.

To measure daily movements, we used three related metrics of local movement: the area of a daily 100% minimum convex polygon (MCP), mean hourly step lengths per day, and mean daily net displacement. Daily MCPs draw a convex hull around all daily locations (i.e., GPS fixes); a larger MCP indicates more movement and more exploratory behavior^72,73^. Mean step length is the average distance between hourly GPS fixes in a day and has been used in prior analyses of influenza in ducks^14,15^. Finally, mean net displacement measures a bird’s daily average distance from its capture location and measures the timing and distance of initial dispersal. We resampled telemetry data to 1-h intervals with a tolerance of 8 min (i.e., GPS fixes between 52 and 68 min apart), then calculated each movement metric per individual per day. We split days at sunrise because ducks usually move between foraging and roosting areas at dawn and dusk^64,73^, so using sunrise as the beginning of a day helps ensure that movement or resting at a single foraging or roosting site are included as part of the same day. We identified sunrise times using statistical software (*suncalc* package version 0.5.0 in R version 4.0.1^74,75^) and calculated MCPs and step lengths (*amt* package version 0.1.4^76^).

For each local movement metric, we fit a linear mixed-effects model with log-transformed area or distance as the response variable (*glmmTMB* package version 1.1.3^77,78^). Explanatory variables were: active influenza infection status at capture (positive or negative); days since influenza sampling; sex; age; and the pairwise interaction between infection status and days since sampling. This interaction was included to test the prediction that movement would change as birds recovered from infection. We log-transformed days since sampling because we expected that differences in movement between infected and uninfected birds would be largest in the first few days following sampling^14,79^. We included log-transformed number of GPS fixes as a fixed effect to account for the sensitivity of movement metrics to sample sizes. We also included an AR1 autoregressive random slope for each individual to account for inter-individual variation and temporal autocorrelation in individuals’ locations over time^80^. We evaluated models using standard plots and tests of residuals (*DHARMa* package version 0.4.3^81^) and calculated post-hoc estimated marginal means and 95% confidence intervals (CIs, *emmeans* package version 1.6.3^82^).

Antibodies from a prior infection can protect birds from the most severe effects of infection, and the presence of antibodies can indicate that an individual is relatively late in its current infection; in either case, we hypothesized that effects of HPAI infection on movement behavior might be smaller in individuals with antibodies to influenza. Therefore, we repeated these models using a combination of active infection and antibody status as a predictor variable. This variable had three levels: HPAI+/antibody+, HPAI+/antibody–, and HPAI– with either antibody status. These models were otherwise identical to the models using active infection status only.

#### Contact rates and environmental transmission

We used observed movement patterns of birds within four days of sampling to identify close and indirect contacts that could have led to transmission. Based on experimental infection data, four days is a conservative estimate of the shedding period for HPAI^27^. We defined a pair of locations as a contact if two birds were observed within 25 m of the same location within 65 min^15^; this 65-min window accounted for different schedules among GPS transmitters, which were not synchronized to provide fixes at the same time as each other, and allowed five minutes for deviations from this hourly schedule. We considered an interaction to be a contact that could lead to transmission if the bird that was present first was infected and the bird that was present second was uninfected.

Next, we examined whether contacts that could have led to transmission were more or less common than would be expected if contacts were random with respect to infection status. To do so, we randomized infection statuses among individuals, then calculated the proportion of contacts that were “possible transmission contacts” in the randomized data. We repeated this process 500 times with replacement, then compared the distribution of proportions in the randomized data to the proportion in the observed data.

We also assessed the potential for environmental transmission of HPAI from GPS-tagged mallards by estimating shared space use between infected and uninfected birds; note that this analysis does not account for the presence of untagged HPAI-positive birds at the site and therefore represents a conservative estimate of environmental transmission. For each infected bird, we calculated a dynamic Brownian bridge movement model (dBBMM^83^; *move* package version 4.0.6^84^) for the first four days following sampling (as above, a conservative estimate of the HPAI shedding period). We used a location error of 23.5 m and a raster resolution of 30 m for dBBMMs. We then extracted the 95% utilization distribution (UD) contour for each infected bird, which represents the area where the infected individual spent 95% of its time during the four-day period. We then defined the “HPAI-contaminated area” for the population, which included any location covered by at least one infected bird’s 95% UD (i.e., the union of the 95% UDs across all infected birds).

Starting at the end of the four-day period for the latest-captured infected bird (February 4, 2022) and continuing until the first date of spring migration (see below; February 11, 2022), we calculated the proportion of time that birds that were uninfected at the time of capture spent inside the HPAI-contaminated area. We started at the end of this period because we had incomplete data on infected birds until the end of this time. For each bird, we calculated the proportion of fixes in the HPAI-contaminated area vs. outside the area for each bird-day. This proportion is a proxy for the daily environmental transmission risk per individual. To understand how this risk varied across individuals, by infection status, over time, and by age or sex, we used a generalized linear mixed-effects model with a logit link to model the proportion of fixes within the contaminated area as a function of days since February 3 (log transformed), HPAI infection status, age, sex, and the interaction between infection status and days since February 3 (using *glmmTMB*^77,78^). We also included an AR1 autoregressive term for each bird^80^, because each bird’s locations on consecutive days are autocorrelated. The model used the number of fixes inside and outside the HPAI-contaminated area as the response variable.

#### Migration patterns

To measure differences in migration phenology and migration patterns between infected and uninfected mallards (Fig. S5), we first segmented each track into wintering, migration, and summer periods. We used bivariate time-series segmentation on latitude and longitude using the *segclust2d* package^85^. This method uses the mean and/or variance in these two variables across the track to identify discrete segments. We visually inspected each track to identify the number of segments that most accurately separated wintering and summering phases from migration and stopover. Because segmentation accurately identifies break points in segments but includes movement bouts with either the previous or subsequent segment, we further segmented tracks by creating a new segment each time a bird was observed moving 20 km/h or faster; this speed was a clear distinction between dispersive (flight) and non-dispersive (local) movements for most birds^86^.

We then classified each segment as winter, migration/stopover, or summer. We defined winter as segments with median locations within 50 km of capture. We defined summer locations as segments lasting at least 30 days and beginning in March-July, with a range of net displacement ≤ 50 km^87,88^. For birds whose transmitters failed before this 30-day period was over, we assigned the last segment as a summer segment if it was at least 1000 km from the capture location and started in March-July. We verified all classifications manually using plots of net displacement over time and maps of the locations of each segment.

From these segmented tracks, we measured four characteristic of each individual’s spring migration: (1) the initiation date of spring migration, i.e., the end date of a bird’s last wintering segment; (2) the duration of spring migration, i.e., the time elapsed between the last day of wintering and the first day of summering; (3) migration distance, i.e., the median net displacement of all summer locations (i.e., median distance from capture site); and (4) migration speed, i.e., migration distance divided by migration duration. For six individuals, it was possible to calculate migration initiation date but not the other metrics because they did not have sufficient tracking data for the full migration period. For each migration metric, we modeled differences between infected and uninfected birds using linear models. Each model used the migration metric as the response variable and included infection status, sex, and age as predictors.

We also developed a separate set of models that measured relationships between these same variables and prior infection (as opposed to active infection status). These models were constructed identically except that infection was measured using bELISA results as well as virus isolation (i.e., active infection) results. We considered an individual as previously infected at the time of migration if it tested positive for antibodies at the time of capture (i.e., a positive bELISA result) or if it was actively infected at the time of capture.

#### Body condition and mortality

We examined differences in body condition at capture between infected and uninfected birds. We estimated body condition using the residuals from a linear regression between body mass (g) and wing chord length (cm), which represent deviation from the expectation of size-adjusted mass in the population^89^. We found no evidence for differences in this relationship by age or sex, so we did not account for age or sex in our calculation of body condition. We tested for differences in body condition between infected and uninfected birds using a linear model with body condition as the response variable and infection status as the predictor variable.

Finally, we evaluated whether survival to the end of the study (October 2022) was related to HPAI infection status at capture. We only included birds confirmed to be dead or alive on October 25, 2022 and omitted birds with unknown fates due to transmitter back-log, lack of cellular connectivity, and/or transmitter failure. We fit a generalized linear model with a logit link that measured mortality as a function of infection status, age, and sex.

### Supplementary Information

Supplementary Information.

## Data Availability

Data are available at USGS ScienceBase (https://doi.org/10.5066/P9AZL1MN)^65^ and code to reproduce all analyses is available at Zenodo (https://doi.org/10.5281/zenodo.8126569)^90^.
