# Supplementary material for: North American wintering mallards infected with highly pathogenic avian influenza show few signs of altered local or migratory movements
Source: Sci Rep. 2023 Sep 2;13:14473. doi: 10.1038/s41598-023-40921-z (PMC10475108; doi:10.1038/s41598-023-40921-z)
Supplement: Supplementary file 1 — Supplementary Information. [file 41598_2023_40921_MOESM1_ESM.docx]

**Supplementary Information**

**North American wintering mallards infected with highly pathogenic avian influenza show few signs of altered local or migratory movements**

Claire S. Teitelbaum^1,2*^, Nicholas M. Masto^3^, Jeffery D. Sullivan^4^, Allison C. Keever^3^, Rebecca L. Poulson^5^, Deborah L. Carter^5^, Abigail G. Blake-Bradshaw^3^, Cory J. Highway^3^, Jamie C. Feddersen^6^, Heath M. Hagy^7^, Richard W. Gerhold^8^, Bradley S. Cohen^3^, Diann J. Prosser^4^

1. Akima Systems Engineering, Herndon, VA, USA
2. Contractor to U.S. Geological Survey, Eastern Ecological Science Center, Laurel, MD, USA
3. College of Arts and Sciences, Tennessee Technological University, Cookeville, TN, USA
4. U.S. Geological Survey, Eastern Ecological Science Center, Laurel, MD, USA
5. Southeastern Cooperative Wildlife Disease Study, College of Veterinary Medicine, University of Georgia, Athens, GA, USA
6. Tennessee Wildlife Resources Agency, Nashville, TN, USA
7. U.S. Fish and Wildlife Service, National Wildlife Refuge System, Stanton, TN, USA
8. University of Tennessee College of Veterinary Medicine, Knoxville, TN, USA

* Corresponding author: Claire Teitelbaum ([claire.teitelbaum@gmail.com](mailto:claire.teitelbaum@gmail.com))

* Current address: Bay Area Environmental Research Institute and NASA Ames Research Center, Moffett Field, CA, USA


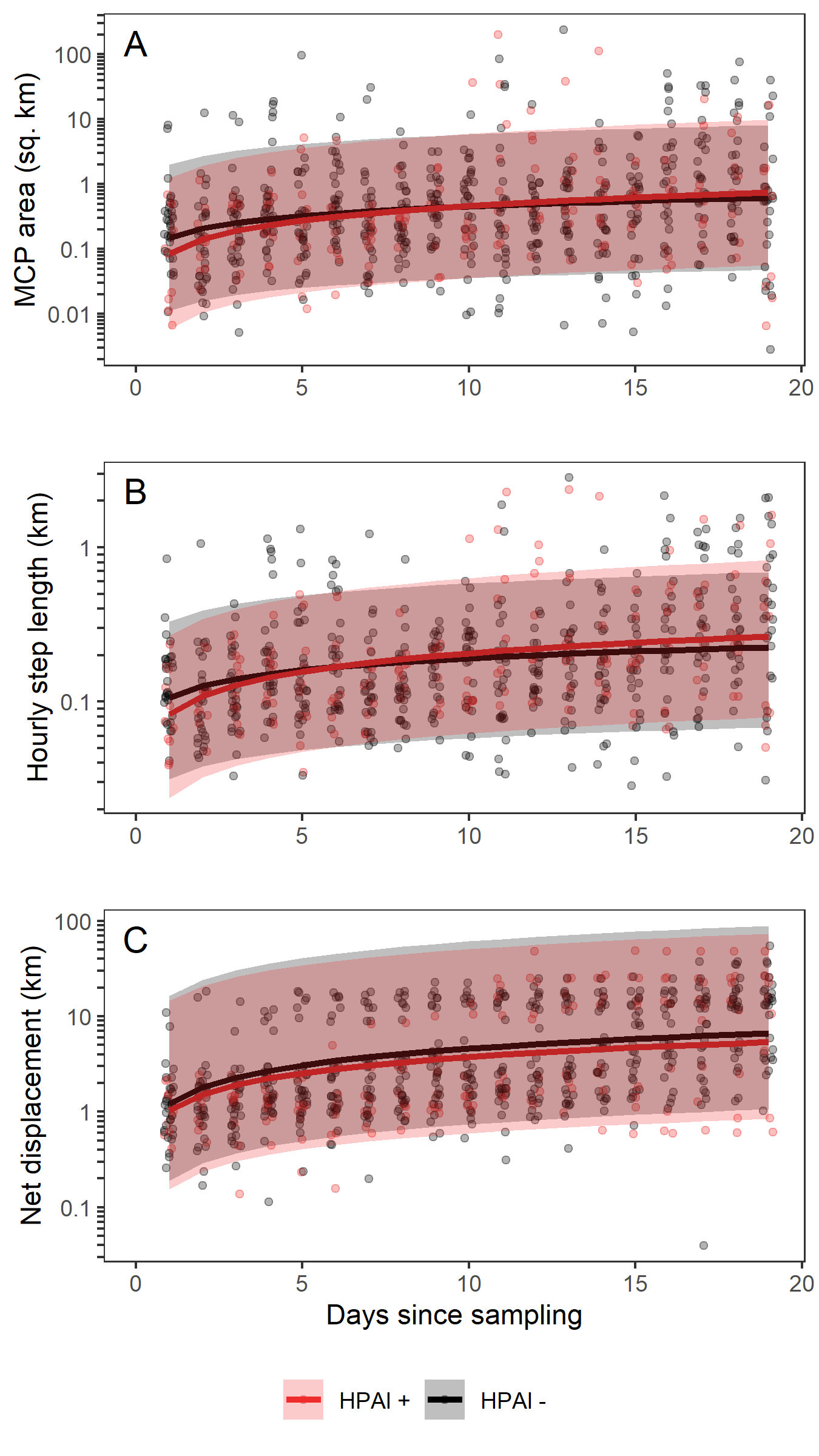


**Figure S1:** Local movement patterns do not differ between mallards (*Anas platyrhynchos*) infected with highly pathogenic avian influenza (HPAI) and uninfected mallards during winter. In each plot, points show raw data, lines show predicted means from a linear mixed-effects model, and shaded areas show 95% confidence intervals of the predictions. Models also included terms for age, sex, and a temporal autoregressive term for each individual; plots show values for an adult female and an “average” individual. (A) Area of a 100% minimum convex polygon (MCP), a measurement of space use. (B) Mean hourly step lengths. (C) Net displacement, i.e., distance from the first GPS fix, a measurement of dispersal from the capture site.


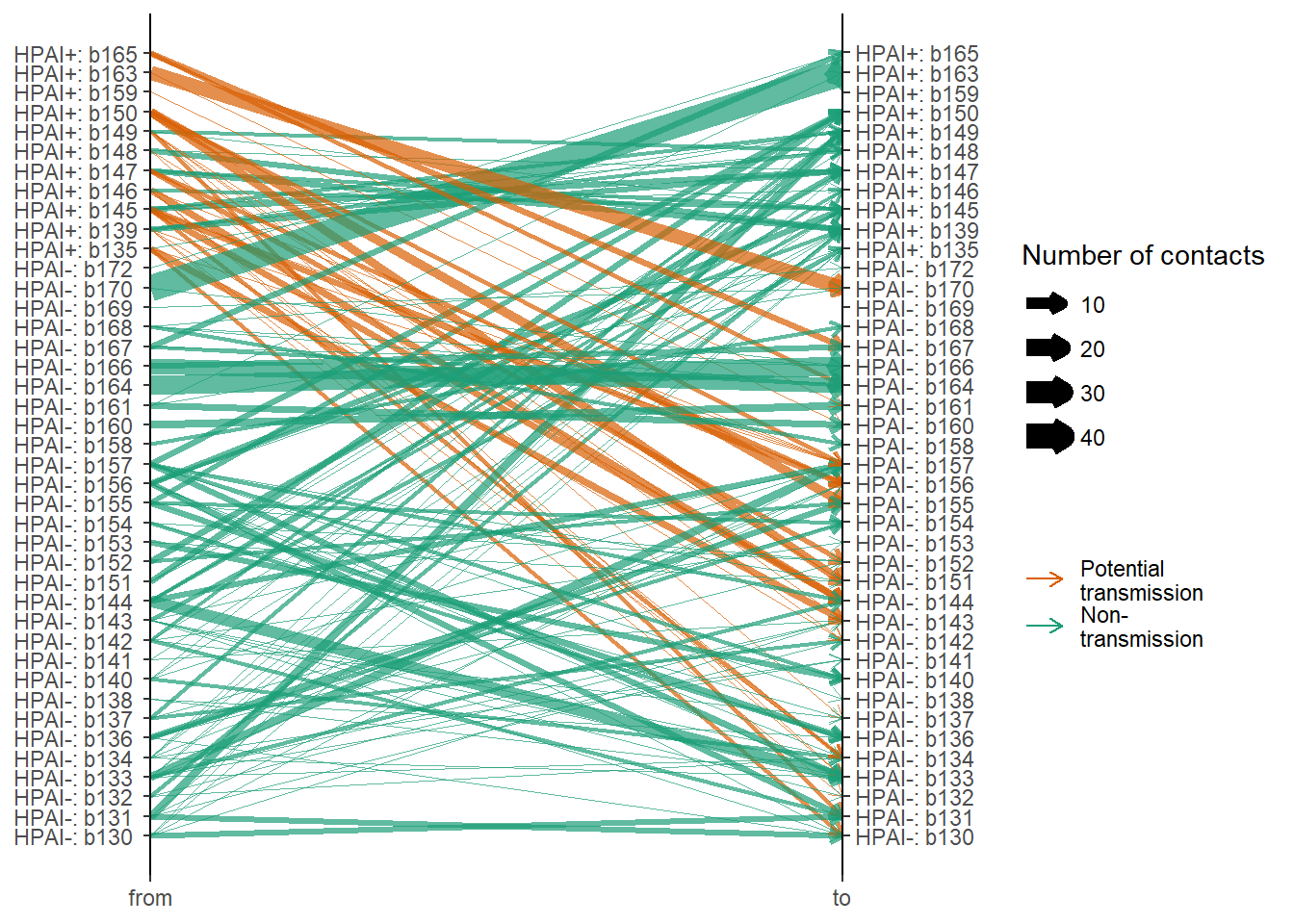


**Figure S2:** Observed contacts between mallards (*Anas platyrhynchos*) marked in this study. Each arrow is an observed relationship between a pair of birds; the thickness of the arrow represents the number of contacts observed during the first four days following sampling for the left-hand individual. Birds were defined as “in contact” if they were within 25 m of one another within 65 minutes; the bird that was present first was defined as the source of the contact. Contacts that could have resulted in influenza transmission (i.e., from an infected to an uninfected bird) are shown in orange.


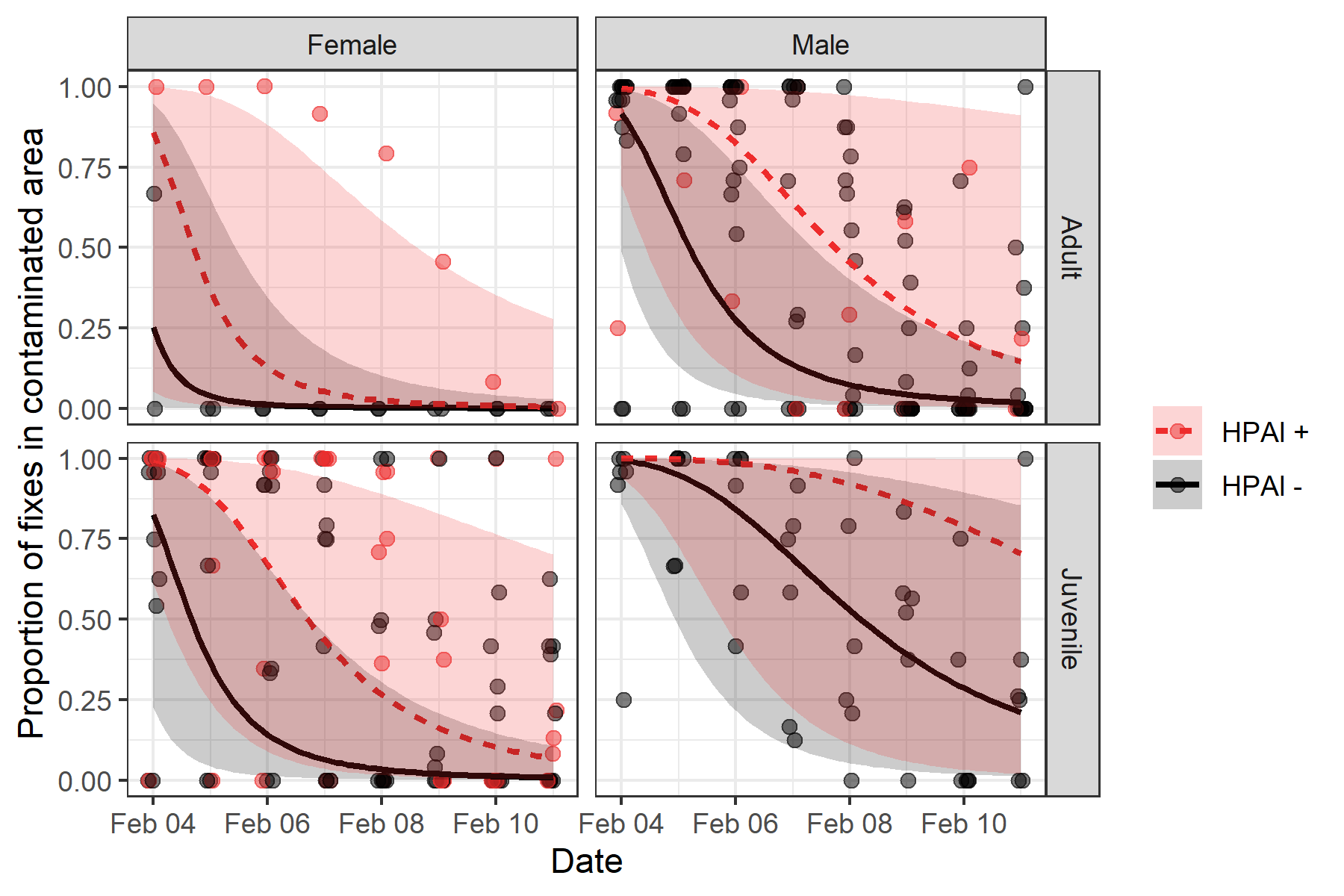


**Figure S3:** Time spent in contaminated habitats by uninfected birds by age and sex. Contaminated habitats were defined as the total area of all 95% utilization distributions of mallards (*Anas platyrhynchos*) infected with highly pathogenic avian influenza (HPAI) in the first four days following sampling. The proportion of time was calculated as the proportion of fixes for each bird within this contaminated area on each day. Points show raw data and are jittered to increase visibility. Each line shows estimated model means from a generalized linear mixed-effects model, which also included an autoregressive term for each individual. Shaded areas show 95% confidence intervals of the estimated mean.


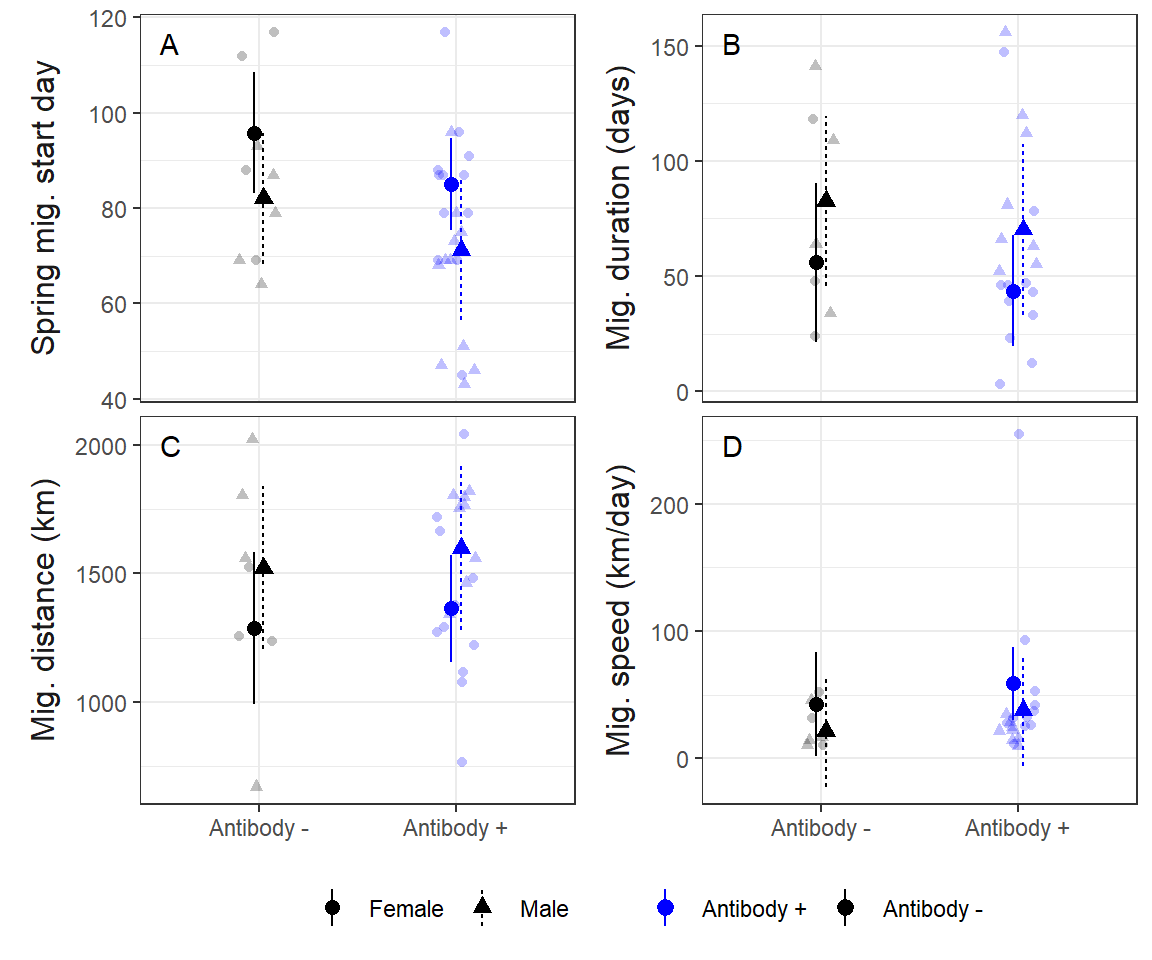


**Figure S4:** Relationships between avian influenza antibody status, sex, and migration patterns in mallards (*Anas platyrhynchos*). Each panel shows the estimated mean and 95% confidence interval of the mean from a linear model. Partially transparent points show raw data. Models also included a term for age; plots show values for juveniles. We found no evidence that migration was related to antibody status for any metric. (A) Spring migration departure date. (B) The duration of migration. (C) Migration distance. (D) Migration speed.


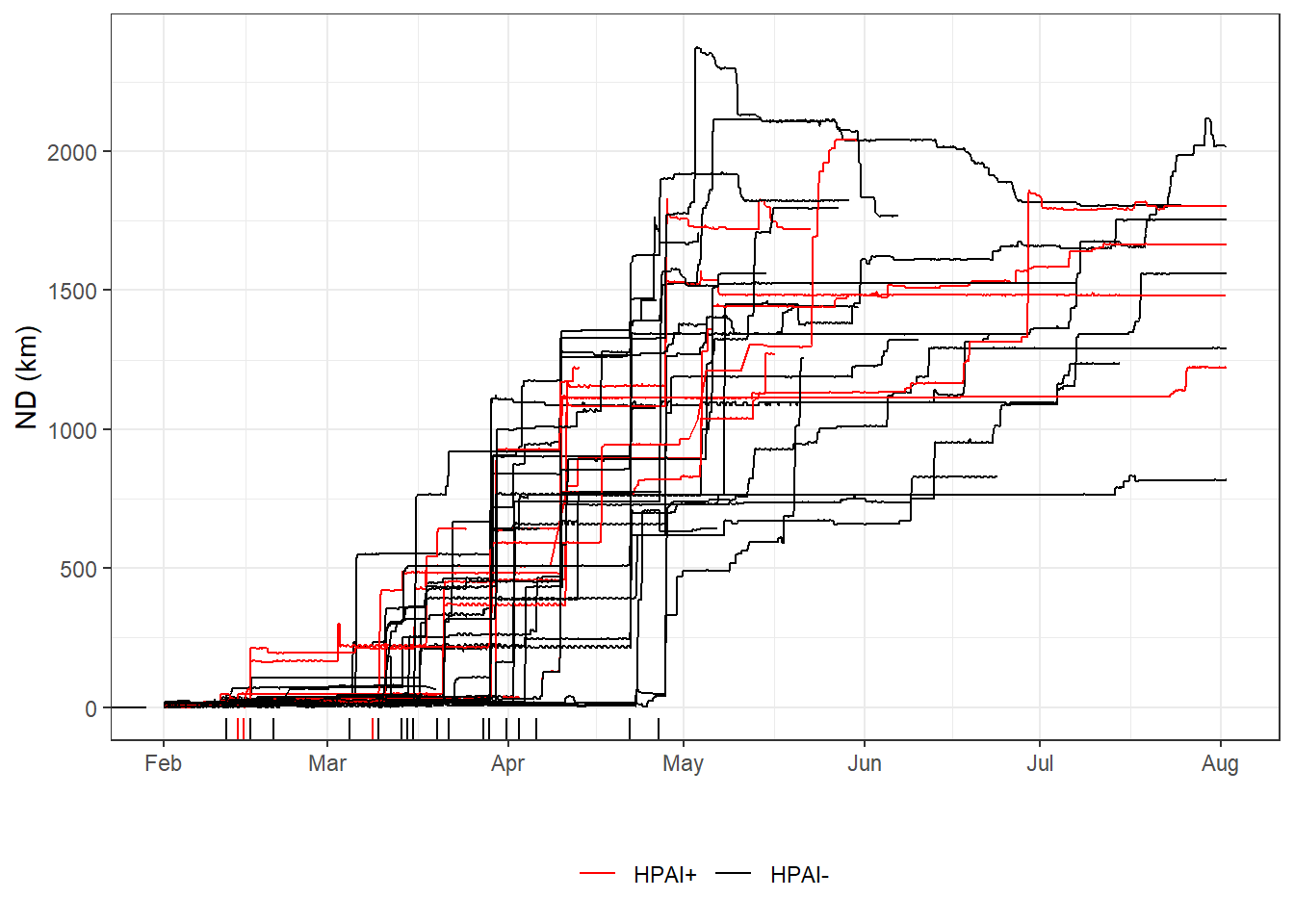
**Figure S5:** Migration patterns in mallards (*Anas platyrhynchos*) infected with highly pathogenic avian influenza (HPAI+, red) and in uninfected mallards (HPAI-, black). Each line shows net displacement (ND, distance from capture location) for an individual from February 1 to August 1 or the end of data availability. Rugplots (lines) at the bottom of the plot show spring migration departure dates for each individual, colored by infection status; some lines overlap.

**Table S1:** Estimated parameters from a model predicting daily log_10_-transformed minimum convex polygon area, a measurement of space use, in mallards (*Anas platyrhynchos*). This model also included an AR1 autoregressive term by individual. The AR standard deviation=0.554, residual (measurement) standard deviation=0.497, autocorrelation=0.593. Uninfected was the reference level for highly pathogenic avian influenza (HPAI) infection.

|  | **Estimate** | **Std. error** | **z value** | **p-value** |
| --- | --- | --- | --- | --- |
| Intercept | -1.806 | 0.525 | -3.437 | 0.001 |
| HPAI infection | -0.241 | 0.225 | -1.074 | 0.283 |
| Days since sampling (log) | 0.211 | 0.053 | 3.992 | <0.001 |
| Age (juvenile) | -0.108 | 0.101 | -1.066 | 0.286 |
| Sex (male) | 0.137 | 0.106 | 1.286 | 0.199 |
| Number of fixes (log) | 0.308 | 0.156 | 1.976 | 0.048 |
| HPAI infection: Days since sampling (log) | 0.108 | 0.103 | 1.050 | 0.294 |

**Table S2:** Estimated parameters from a model predicting daily log_10_-transformed mean hourly step length, a measurement of daily movement, in mallards (*Anas platyrhynchos*). The AR standard deviation=0.249, residual (measurement) standard deviation=0.231, autocorrelation=0.654. Uninfected was the reference level for highly pathogenic avian influenza (HPAI) infection.

|  | **Estimate** | **Std. error** | **z value** | **p-value** |
| --- | --- | --- | --- | --- |
| Intercept | -0.677 | 0.279 | -2.429 | 0.015 |
| HPAI infection | -0.105 | 0.106 | -0.989 | 0.323 |
| Days since sampling (log) | 0.111 | 0.026 | 4.366 | <0.001 |
| Age (juvenile) | -0.043 | 0.051 | -0.856 | 0.392 |
| Sex (male) | 0.069 | 0.052 | 1.322 | 0.186 |
| Number of fixes (log) | -0.099 | 0.083 | -1.193 | 0.233 |
| HPAI infection: Days since sampling (log) | 0.059 | 0.048 | 1.231 | 0.218 |

**Table S3:** Estimated parameters from a model predicting daily log_10_-transformed mean daily net displacement, a measurement of dispersal, in mallards (*Anas platyrhynchos*). The AR standard deviation=0.473, residual (measurement) standard deviation=0.00006, autocorrelation=0.824; note the high temporal autocorrelation in this variable. Uninfected was the reference level for highly pathogenic avian influenza (HPAI) infection.

|  | **Estimate** | **Std. error** | **z value** | **p-value** |
| --- | --- | --- | --- | --- |
| Intercept | -0.262 | 0.203 | -1.292 | 0.196 |
| HPAI infection | -0.068 | 0.167 | -0.408 | 0.684 |
| Days since sampling (log) | 0.251 | 0.037 | 6.850 | <0.001 |
| Age (juvenile) | -0.199 | 0.108 | -1.849 | 0.064 |
| Sex (male) | -0.108 | 0.113 | -0.959 | 0.338 |
| Number of fixes (log) | 0.161 | 0.046 | 3.513 | <0.001 |
| HPAI infection: Days since sampling (log) | -0.008 | 0.072 | -0.115 | 0.908 |

**Table S4:** Estimated parameters from a model predicting daily log_10_-transformed minimum convex polygon area, in mallards (*Anas platyrhynchos*), using both active infection and antibody data. The AR standard deviation=0.554, residual (measurement) standard deviation=0.497, autocorrelation=0.590. Uninfected (with or without antibodies to avian influenza) was the reference level for infection status.

|  | **Estimate** | **Std. error** | **z value** | **p-value** |
| --- | --- | --- | --- | --- |
| Intercept | -1.802 | 0.526 | -3.428 | 0.001 |
| HPAI+/antibody– | -0.247 | 0.308 | -0.802 | 0.422 |
| HPAI+/antibody+ | -0.236 | 0.286 | -0.824 | 0.410 |
| Days since sampling (log) | 0.211 | 0.053 | 3.993 | <0.001 |
| Age (juvenile) | -0.112 | 0.103 | -1.086 | 0.277 |
| Sex (male) | 0.135 | 0.106 | 1.266 | 0.205 |
| Number of fixes (log) | 0.308 | 0.156 | 1.975 | 0.048 |
| HPAI+/antibody–: Days since sampling (log) | 0.100 | 0.142 | 0.707 | 0.480 |
| HPAI+/antibody+: Days since sampling (log) | 0.114 | 0.130 | 0.874 | 0.382 |

**Table S5:** Estimated parameters from a model predicting the proportion of time spent in contaminated habitats by mallards (*Anas platyrhynchos*) prior to the initiation of migration. The AR standard deviation=5.085, autocorrelation=0.862.

|  | **Estimate** | **Std. error** | **z value** | **p-value** |
| --- | --- | --- | --- | --- |
| Intercept | -1.081 | 2.025 | -0.534 | 0.593 |
| Days since sampling (log) | -3.056 | 0.545 | -5.610 | <0.001 |
| HPAI infection | 2.879 | 2.280 | 1.263 | 0.207 |
| Age (juvenile) | 2.637 | 1.662 | 1.587 | 0.113 |
| Sex (male) | 3.475 | 1.772 | 1.961 | 0.050 |
| HPAI infection: Days since sampling (log) | -0.333 | 1.175 | -0.283 | 0.777 |

**Table S6:** Results from linear models predicting characteristics of migration from active highly pathogenic avian influenza (HPAI) infection status in mallards (*Anas platyrhynchos*). Adults were the reference level for age, females were the reference level for sex, and uninfected was the reference level for infection status.

|  | **Start date** | | | | **Duration** | | | | **Distance** | | | | **Speed** | | | | |  |
| --- | --- | --- | --- | --- | --- | --- | --- | --- | --- | --- | --- | --- | --- | --- | --- | --- | --- | --- |
|  | Est-imate | Std. error | t-value | p-value | Est-imate | Std. error | t-value | p-value | Estimate | Std. error | t-value | p-value | | Est-imate | Std. error | t-value | p-value | |
| **Intercept** | 82.654 | 7.227 | 11.437 | <0.001 | 55.790 | 18.182 | 3.068 | 0.005 | 1311.787 | 155.176 | 8.454 | <0.001 | | 49.832 | 21.378 | 2.331 | 0.028 | |
| **Infection status** | -13.180 | 6.574 | -2.005 | 0.054 | 14.403 | 17.498 | 0.823 | 0.418 | 228.290 | 149.339 | 1.529 | 0.139 | | -16.734 | 20.574 | -0.813 | 0.424 | |
| **Age** | 10.081 | 6.472 | 1.558 | 0.129 | -15.139 | 16.478 | -0.919 | 0.367 | -43.915 | 140.630 | -0.312 | 0.757 | | 11.130 | 19.374 | 0.574 | 0.571 | |
| **Sex** | -14.379 | 6.796 | -2.116 | 0.043 | 33.292 | 17.719 | 1.879 | 0.072 | 265.168 | 151.221 | 1.754 | 0.092 | | -30.406 | 20.833 | -1.459 | 0.157 | |

**Table S7:** Results from linear models predicting characteristics of migration from avian influenza antibody status in mallards (*Anas platyrhynchos*). Adults were the reference level for age, females were the reference level for sex, and antibody-negative was the reference level for antibody status.

|  | **Start date** | | | | **Duration** | | | | **Distance** | | | | **Speed** | | | | |  |
| --- | --- | --- | --- | --- | --- | --- | --- | --- | --- | --- | --- | --- | --- | --- | --- | --- | --- | --- |
|  | Est-imate | Std. error | t-value | p-value | Est-imate | Std. error | t-value | p-value | Estimate | Std. error | t-value | p-value | | Est-imate | Std. error | t-value | p-value | |
| **Intercept** | 88.033 | 10.189 | 8.640 | <0.001 | 76.096 | 25.244 | 3.014 | 0.006 | 1348.117 | 217.502 | 6.198 | <0.001 | | 26.664 | 29.972 | 0.890 | 0.383 | |
| **Antibody status** | -10.826 | 7.046 | -1.536 | 0.136 | -12.341 | 18.357 | -0.672 | 0.508 | 76.374 | 158.168 | 0.483 | 0.634 | | 16.334 | 21.796 | 0.749 | 0.462 | |
| **Age** | 7.799 | 7.376 | 1.057 | 0.299 | -20.236 | 18.357 | -1.102 | 0.282 | -61.058 | 158.168 | -0.386 | 0.703 | | 16.012 | 21.796 | 0.735 | 0.470 | |
| **Sex** | -13.777 | 7.060 | -1.951 | 0.061 | 26.627 | 18.095 | 1.472 | 0.155 | 235.249 | 155.903 | 1.509 | 0.146 | | -21.228 | 21.484 | -0.988 | 0.334 | |
